# Validity of Administrative Databases in Comparison to Medical Charts for Breast Cancer Treatment Data

**DOI:** 10.1155/2018/9218595

**Published:** 2018-05-14

**Authors:** Ashini Weerasinghe, Courtney R. Smith, Vicky Majpruz, Anjali Pandya, Kristina M. Blackmore, Claire M. B. Holloway, Roanne Segal-Nadlere, Cathy Paroschy Harris, Ashley Hendry, Amanda Hey, Anat Kornecki, George Lougheed, Barbara-Anne Maier, Patricia Marchand, David McCready, Carol Rand, Simon Raphael, Neelu Sehgal, Anna M. Chiarelli

**Affiliations:** ^1^Prevention and Cancer Control, Cancer Care Ontario, 620 University Avenue, Toronto, ON, Canada M5G 2L7; ^2^Dalla Lana School of Public Health, University of Toronto, 155 College Street, 6th Floor, Toronto, ON, Canada M5T 3M7; ^3^Sunnybrook Health Sciences Centre, 2075 Bayview Avenue, Toronto, ON, Canada M4N 3M5; ^4^Department of Surgery, University of Toronto, 149 College Street, 5th Floor, Toronto, ON, Canada M5T 1P5; ^5^Champlain Regional Cancer Program, 501 Smyth Road, Ottawa, ON, Canada K1H 8L6; ^6^Prevention and Screening Services, Northwestern Ontario, 980 Oliver Road, Thunder Bay, ON, Canada P7B 6V4; ^7^South East Regional Cancer Program, 25 King Street West, Kingston, ON, Canada K7L 5P9; ^8^North East Regional Cancer Program, 41 Ramsey Lake Road, Sudbury, ON, Canada P3E 5J1; ^9^South West Regional Cancer Program, 790 Commissioners Road East, London, ON, Canada N6A 4L6; ^10^North Simcoe Muskoka Regional Cancer Program, 201 Georgian Drive, Barrie, ON, Canada L4M 6M2; ^11^Waterloo Wellington Regional Cancer Program, 835 King Street West, Kitchener, ON, Canada N2G 1G3; ^12^Central East Regional Cancer Program, 1 Hospital Court, Oshawa, ON, Canada L1G 2B9; ^13^Princess Margaret Cancer Centre, 610 University Ave, Toronto, ON, Canada M5G 2M9; ^14^Hamilton Niagara Haldimand Brant Regional Cancer Program, 699 Concession Street, Hamilton, ON, Canada L8V 5C2; ^15^Central Regional Cancer Program, 596 Davis Drive, Newmarket, ON, Canada L3Y 2P9; ^16^Erie St. Clair Regional Cancer Program, 2220 Kildare Road, Windsor, ON, Canada N8W 2X3

## Abstract

**Objective:**

Medical chart abstraction is the gold standard for collecting breast cancer treatment data for monitoring and research. A less costly alternative is the use of administrative databases. This study will evaluate administrative data in comparison to medical charts for breast cancer treatment information.

**Study Design and Setting:**

A retrospective cohort design identified 2,401 women in the Ontario Breast Screening Program diagnosed with invasive breast cancer from 2006 to 2009. Treatment data were obtained from the Activity Level Reporting and Canadian Institute of Health Information databases. Medical charts were abstracted at cancer centres. Sensitivity, specificity, positive and negative predictive value, and kappa were calculated for receipt and type of treatment, and agreement was assessed for dates. Logistic regression evaluated factors influencing agreement.

**Results:**

Sensitivity and specificity for receipt of radiotherapy (92.0%, 99.3%), chemotherapy (77.7%, 99.2%), and surgery (95.8%, 100%) were high but decreased slightly for specific radiotherapy anatomic locations, chemotherapy protocols, and surgeries. Agreement increased by radiotherapy year (trend test, *p* < 0.0001). Stage II/III compared to stage I cancer decreased odds of agreement for chemotherapy (OR = 0.66, 95% CI: 0.48–0.91) and increased agreement for partial mastectomy (OR = 3.36, 95% CI: 2.27–4.99). Exact agreement in treatment dates varied from 83.0% to 96.5%.

**Conclusion:**

Administrative data can be accurately utilized for future breast cancer treatment studies.

## 1. Introduction

Breast cancer is the most frequently diagnosed cancer among women in Ontario and is the second leading cause of cancer death [1]. Abstraction of medical charts is considered the gold standard for collecting breast cancer treatment data for monitoring and research purposes. However, this process can be laborious and costly, especially when conducting large population-based epidemiology research [2]. An alternative method is the use of administrative databases.

Previous studies using US data have revealed that the overall agreement between medical charts and administrative data for breast cancer treatment type is high, but agreement for specific treatment types and dates has not been evaluated concurrently [3–10]. Only one study has examined receipt of breast cancer radiotherapy in administrative databases in comparison to medical charts [9] but there have been no studies that have examined the validity of the anatomic location to which radiotherapy was received or the validity of radiotherapy start and end dates. Three US studies have examined the validity of breast cancer chemotherapy protocols [6, 7, 10] and found that they were of high accuracy. One study has validated chemotherapy dates and found moderate agreement with medical charts [8]. Validity of breast cancer surgery data in administrative databases has been examined in Ontario [11], demonstrating 86.2% agreement with medical charts; however, this includes a small cohort from the early 1990s and only validated very broad categories of surgery. A more recent study evaluated agreement for breast cancer surgery between hospital records, medical claims, and the cancer registry in Manitoba and found substantial or almost perfect agreement between data sources [12]. Surgery dates, however, were not validated in either study. Several studies have examined factors associated with agreement between administrative data and medical charts and found that agreement decreased with age, stage, and diagnosis year and varied by treatment site [3–5, 7, 8].

The Activity Level Reporting (ALR) database is housed at Cancer Care Ontario and collects selected systemic therapy and all radiation treatment from regional cancer centres and their associated hospitals [13]. Admission and discharge information for surgeries in Ontario is collected by the Canadian Institute of Health Information's hospital abstracting databases (Discharge Abstract Database [DAD], National Ambulatory Care and Reporting System [NACRS]). The purpose of this study was to evaluate the validity of the ALR and CIHI's DAD and NACRS databases in comparison to medical charts for breast cancer treatment data and to examine factors that may influence agreement. Specifically, the validity of the ALR was evaluated for the receipt of radiotherapy and chemotherapy and for specific radiotherapy anatomic locations and chemotherapy protocols and their corresponding treatment dates. CIHI's DAD and NACRS databases were evaluated for the receipt of surgery and for specific surgery types and their corresponding dates.

## 2. Methods

### 2.1. Selection of Breast Cancer Cases

The Ontario Breast Screening Program (OBSP) is a province-wide, organized screening program that provides high-quality breast cancer screening services for women aged 50 to 74 [14]. Women are not eligible if they have acute breast symptoms, a history of breast cancer, or current breast implants [14]. This study identified women aged 50–69 screened through the OBSP between January 1, 2006, and December 31, 2009, with an abnormal mammogram and a diagnosis of screen-detected invasive breast cancer. Exclusions included prevalent cancers detected on initial screens, premenopausal women, bilateral or nonprimary breast cancer, non-Ontario residents, diagnoses more than 1 year following abnormal screening, stage IV breast cancer, women who were missing information required to identify treatment centre location, and women who received treatment at a hospital with less than 15 eligible women.

### 2.2. Data Collection

#### 2.2.1. Medical Chart Abstraction

Trained chart abstractors visited regional cancer centres across Ontario and reviewed medical charts to abstract relevant treatment data for all eligible women. Regional cancer centres are specialized centres in Ontario that deliver all cancer radiotherapy, and patients may also be referred to a regional cancer centre for diagnostic work-up, systemic therapy, and/or treatment planning. These centres and their associated hospitals maintain detailed patient charts about breast cancer treatment information and they have been shown to have complete, high-quality information [15, 16]. To facilitate chart abstraction, a local collaborator was identified for each regional cancer centre. A chart abstraction form was developed to collect demographic, prognostic, and treatment data. Abstraction occurred between 2014 and 2016.

Age at diagnosis and year of diagnosis were recorded. Women's postal code of residence at screening was linked to the 2006 Canadian Census to determine income quintiles (Q1 (low)–Q5 (high)) and community status [17]. Community status included urban (population 10,000+), rural (<10,000 and a strong Metropolitan Influenced Zone (MIZ)), rural remote (<10,000 and a moderate MIZ), and rural very remote (<10,000 and a weak/no MIZ) [17]. Women were categorized as having no comorbidities if they had no preexisting illnesses other than arthritis or high blood pressure at the time of diagnosis and comorbid if they had any other preexisting illness at diagnosis outlined by the Charlson Index [18]. Breast cancer classification (invasive without associated ductal carcinoma in situ or invasive with associated ductal carcinoma in situ) was also recorded. Stage (I, II, and III) was based on the TNM classification scheme (6th edition) [19] and tumour grade was categorized as 1, 2, or 3. Women with negative results for estrogen and progesterone receptors (i.e., immunohistochemical assays showing <1% of tumour cells positive for antibody nuclear staining) [20] and negative for HER2/neu protein overexpression (score 0, 1+) [21] were categorized as triple negative. Treatment centre region (South Central, South Eastern, South Western, and Northern [22]) was classified according to the regional cancer centre a woman first attended. Radiotherapy data included the anatomic location of radiotherapy given, the start and end dates of treatment, and whether treatment was completed. Chemotherapy data included the chemotherapy protocol given, the start and end dates of treatment, and whether treatment was completed. Surgery data included the type of surgery performed and the date of the surgery.

#### 2.2.2. Administrative Data

Administrative data on radiotherapy and chemotherapy were obtained from the ALR. The ALR includes data submitted to Cancer Care Ontario by regional cancer centres and their associated hospitals. Radiotherapy data included the visit date of the activity, disease site, and the anatomic location of the body that received treatment. Chemotherapy data included the visit date of the activity, disease site, and medications given during chemotherapy.

Administrative data on surgery were obtained from CIHI's DAD and NACRS databases. DAD is a health services database that receives inpatient hospital discharge data directly from Ontario hospitals. NACRS is a health services database that receives ambulatory hospital and clinic discharge data from hospitals in Ontario. Surgery data included the date of the procedure, the type of procedure, and the main reason for the procedure (e.g., breast cancer).

### 2.3. Data Analysis

Medical chart data was linked with the ALR and CIHI databases using the Ontario Cancer Registry group number. For ALR radiotherapy data, a record was excluded if it indicated a non-breast disease site, if the treatment end date was more than 3 months after the follow-up chart abstraction date or more than 18 months after the diagnosis date (as these records likely indicate treatment for a second primary tumour or metastasis), if it was within 18 months but related to a recurrence, or if it indicated an incomplete treatment. For ALR chemotherapy data, a record was excluded if it indicated a non-breast disease site, if treatment end date was more than 3 months after follow-up chart abstraction date or more than 24 months after the diagnosis date, if it indicated receipt of hormone therapy only, or if it indicated that the chemotherapy protocol was unknown. For CIHI surgery data, a record was excluded if it was unrelated to invasive breast cancer, if it was missing surgery date and type, if the surgery was not treatment related, or if the surgery occurred before diagnosis or more than 12 months after diagnosis.

Sensitivity, specificity, positive predictive value (PPV), negative predictive value (NPV), and kappa statistics were calculated for the receipt of radiotherapy, chemotherapy, and surgery, as well as for specific radiotherapy anatomic locations, chemotherapy protocols, and surgery types. The kappa statistic accounts for chance agreement and was classified into five levels: slight agreement (<0.20), fair agreement (0.21–0.40), moderate agreement (0.41–0.60), substantial agreement (0.61–0.80), and almost perfect agreement (0.81–1.00) [23, 24]. To compare radiotherapy anatomic locations, analyses were restricted to women that received radiotherapy according to medical charts. Bilateral internal mammary chain was grouped with the supraclavicular/axilla region and radiation to the breast and chest wall were grouped. To compare chemotherapy protocols, analyses were restricted to women that received chemotherapy according to medical charts and had only one chemotherapy protocol. Agreement analyses for chemotherapy protocols were further restricted to exclude women that received a clinical trial according to the ALR, as it was not noted which specific drug(s) they received. To compare surgery types, analyses were restricted to women that received surgery according to medical charts. Percent agreement for radiotherapy and chemotherapy start and end dates and surgery dates in medical charts and administrative databases was also calculated for an exact match, ±1 day and ±7 days. Dates were validated by type for the most frequent treatment type and restricted to those women with treatment types that matched between the medical charts and the administrative data.

Multivariable logistic regression estimated odds ratios (OR) and 95% confidence intervals (CI) for factors influencing agreement between administrative databases and medical charts. Age at diagnosis (50–59, 60–69), year of diagnosis (2006, 2007, 2008, and 2009), TNM stage (I, II/III), and treatment centre region (South Central, South Eastern, South Western, and Northern) were adjusted for in all models. An offset controlling for agreement due to chance was included in all models [25]. Regression analyses were conducted for the receipt of each therapy and for the most common treatment type for each therapy. All analyses were performed using SAS version 9.4 [26]. The study was approved by the University of Toronto Research Ethics Board and informed consent was not required.

## 3. Results

**Figure 1:**
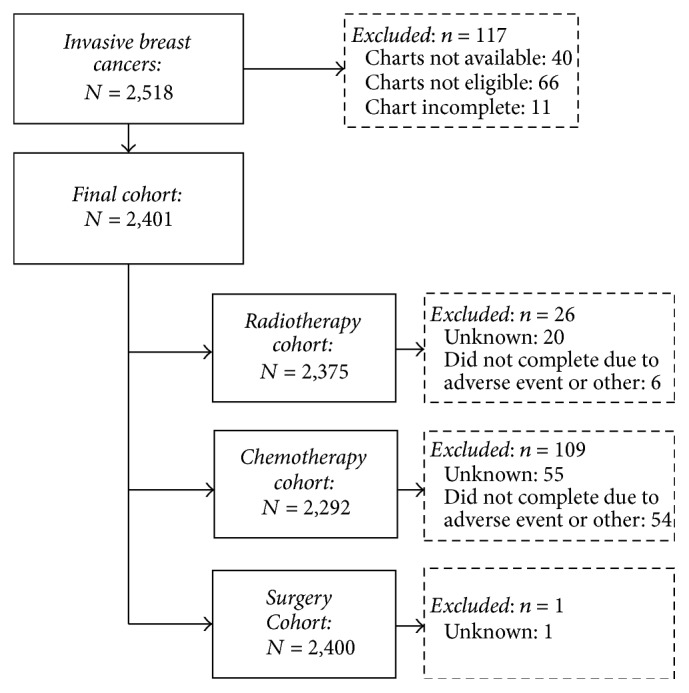
Cohort of women aged 50–69 diagnosed with screen-detected invasive breast cancer within the Ontario Breast Screening Program between January 1, 2006, and December 31, 2009.

Overall, 2,518 eligible women diagnosed with stages I–III, unilateral, screen-detected invasive breast cancer were identified (Figure 1). Women were excluded if their medical charts were not available (*n* = 40), not eligible after review (*n* = 66), or incomplete (*n* = 11). The final sample consisted of 2,401 women of whom 2,375 had complete radiotherapy data, 2,292 had complete chemotherapy data, and 2,400 had complete surgery data.

**Figure 2:**
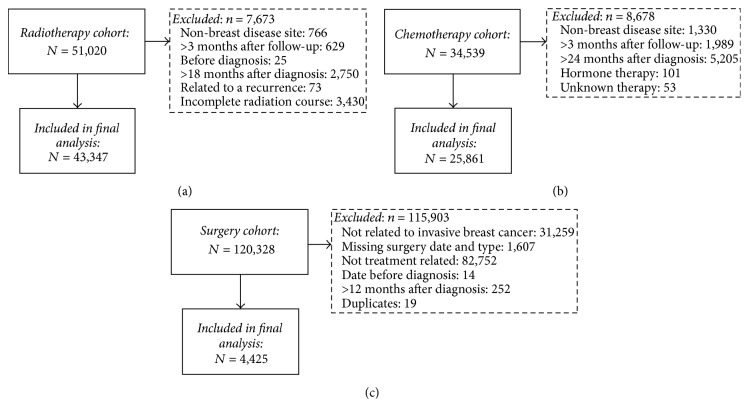
Selection of (a) radiotherapy records and (b) chemotherapy records within the Activity Level Reporting database and (c) surgery records within the Canadian Institute for Health Information databases.

There were a total of 51,020 records in the ALR for the 2,375 women in the radiotherapy cohort (Figure 2(a)). Records were excluded if they indicated a non-breast disease site (*n* = 766), if the treatment end date was more than 3 months after follow-up chart abstraction date (*n* = 629), if the treatment end date was before diagnosis (*n* = 25) or more than 18 months after the diagnosis (*n* = 2,750), if treatment was within 18 months of diagnosis but was related to a recurrence (*n* = 73), or if the radiation course was incomplete (*n* = 3,430). There were a total of 34,539 records in the ALR for the 2,292 women in the chemotherapy cohort (Figure 2(b)). Records were excluded if they indicated a non-breast disease site (*n* = 1,330), if the treatment end date was more than 3 months after follow-up chart abstraction date (*n* = 1,989) or more than 24 months after diagnosis (*n* = 5,205), if the records were for hormone therapy only (*n* = 101), or if the chemotherapy protocol was unknown (*n* = 53). There were a total of 120,328 records in the CIHI databases for the 2,400 women in the surgery cohort (Figure 2(c)). Records were excluded if they were not related to invasive breast cancer (*n* = 31,259), were missing surgery date and type (*n* = 1,607), were not relevant for breast cancer treatment (*n* = 82,752), occurred before diagnosis (*n* = 14) or more than 12 months after diagnosis (*n* = 252), or were duplicates between the CIHI DAD and NACRS databases (*n* = 19).

Women with complete treatment information for radiotherapy, chemotherapy, and surgery represent overlapping cohorts and thus have very similar characteristics (Table 1). Approximately two-thirds of women were aged 60 to 69 and one-third were diagnosed in 2009. The majority were from an urban community setting and approximately one-quarter were from the highest income quintile. More than half of the women did not report any comorbidities, and most were classified as having invasive breast cancer with associated ductal carcinoma in situ. Approximately two-thirds of women had stage I breast cancer, with half having an intermediate grade tumour. Most patients did not have triple negative hormone status. More than half of the women received treatment in the South Central region of Ontario, which represents the Greater Toronto Area.

For receipt of radiotherapy, sensitivity was 92.0%, specificity was 99.3%, PPV was 99.8%, and NPV was 71.9% (Table 2). The kappa statistic showed substantial agreement between medical charts and the ALR (0.793). Sensitivity was high for breast/chest wall radiation (88.2%) and supraclavicular/axilla radiation (86.6%) but lower for breast boost (43.3%), while specificity was greater than 98% for both breast boost and supraclavicular/axilla radiation. PPV was more than 90% for all radiotherapy anatomic locations and NPV was more than 83% for all radiotherapy anatomic locations. The kappa statistic showed moderate agreement for breast boost (kappa = 0.520) and almost perfect agreement for supraclavicular/axilla radiation (kappa = 0.868).

For receipt of chemotherapy, sensitivity was 77.7%, specificity was 99.2%, PPV was 98.2%, and NPV was 88.5% (Table 2). The kappa statistic showed substantial agreement between medical charts and the ALR (0.804). Sensitivity was high for the chemotherapy protocol Fluorouracil, Epirubicin, Cyclophosphamide, Docetaxel (FEC-D; 71.9%) but lower for all others (65.3% for Adriamycin, Cyclophosphamide, Paclitaxel [ACP]/Adriamycin, Cyclophosphamide, Taxol [ACT], 62.5% for Fluorouracil, Epirubicin, Cyclophosphamide [FEC], 60.6% for Adriamycin, Cyclophosphamide [AC], and 50.7% for Taxotere, Cyclophosphamide or Carboplatin [TC]), while specificity was greater than 94% for all chemotherapy protocols. PPV was more than 90% for all chemotherapy protocols, except for TC, which was 72.3%. The NPV for all chemotherapy protocols was above 80%. The kappa statistic showed substantial agreement for all chemotherapy protocols (kappa = 0.682 for FEC-D, 0.714 for AC, 0.733 for ACP/ACT, and 0.734 for FEC), except for TC, which showed moderate agreement (kappa = 0.559).

For receipt of surgery, sensitivity was 95.8%, and specificity and PPV were both 100% (Table 2). Sensitivity was high for partial mastectomy (94.2%) and modified radical mastectomy (89.5%), but lower for sentinel lymph node biopsy (73.7%), simple total mastectomy (60.7%), and axillary node dissection (27.4%). Specificity was very high for all surgery types (partial mastectomy, 91.7%; modified radical mastectomy, 98.3%; simple total mastectomy, 99.1%; axillary node dissection, 98.7%) but lower for sentinel lymph node biopsy (71.9%). PPV was greater than 86% for all surgery types; however, NPV ranged from 53.6% for sentinel lymph node biopsy to 98.6% for modified radical mastectomy. Kappa statistics varied by surgery type and showed fair agreement (axillary node dissection, 0.314), moderate agreement (sentinel lymph node biopsy, 0.415), substantial agreement (simple total mastectomy, 0.693; partial mastectomy, 0.781), and almost perfect agreement (modified radical mastectomy, 0.869).

For receipt of radiotherapy, odds of chance-corrected agreement were 1.41 times higher for women aged 60–69 compared to women aged 50–59 (OR = 1.41, 95% CI: 1.00–1.99; Table 3). For year of diagnosis, odds of chance-corrected agreement for radiotherapy were significantly higher for subsequent years compared to 2006 (OR = 1.59, 95% CI: 1.05–2.42 for 2007; OR = 1.81, 95% CI: 1.16–2.83 for 2008; OR = 3.72, 95% CI: 2.12–6.54 for 2009; test for trend *p* < 0.0001). No factors were associated with agreement for breast or chest wall radiation. For receipt of chemotherapy, odds of chance-corrected agreement were 1.94 times higher for diagnoses in 2008 (OR = 1.94, 95% CI: 1.20–3.16) and 34% lower for stage II/III breast cancer compared to stage I (OR = 0.66, 95% CI: 0.48–0.91). Odds of chance-corrected agreement for surgery overall could not be calculated, due to limited variability in receipt of surgery. For partial mastectomy, odds of chance-corrected agreement were 3.36 times higher for stage II/III breast cancer compared to stage I (OR = 3.36, 95% CI: 2.27–4.99).

Agreement in treatment dates was also examined among those women with matching treatment types between medical charts and administrative data (Table 4). Among 1,623 women with matching breast/chest wall radiation in medical charts and the ALR, 96.5% of start dates were an exact match, increasing to 98.7% for ±7 days. Agreement for end dates was slightly lower, with 83.0% dates matching exactly, increasing to 95.4% for ±7 days. Among 207 women with matching FEC-D chemotherapy protocols between medical charts and the ALR, 88.7% of start dates were an exact match, increasing to 96.1% for ±7 days. Agreement for end dates was slightly lower, with 85.4% dates matching exactly, increasing to 94.7% for ±7 days. Among 1,917 women with matching partial mastectomy surgeries between medical charts and CIHI databases, exact agreement for dates was very high at 96.3%, increasing to 98.2% for ±7 days.

## 4. Discussion

This study found that sensitivity, specificity, PPV, NPV, and kappa were high for receipt of radiotherapy, chemotherapy, and surgery. Agreement decreased slightly when considering specific radiotherapy anatomic locations, chemotherapy protocols, and surgery types. Odds of chance-corrected agreement tended to increase with more recent diagnosis year and were impacted by stage of treatment. Approximately 95% of start and end dates for radiotherapy and chemotherapy and surgery dates in administrative databases were within a week of the dates recorded in the medical charts.

Agreement for receipt of radiotherapy overall was substantial. Sensitivity and PPV were high for breast/chest wall radiation; however specificity and NPV could not be reliably calculated due to minimal variability, as almost all women received this type of radiation. Agreement was also very high for supraclavicular/axilla radiation, but there was lower sensitivity and only moderate agreement for breast boost. Moderate agreement for breast boost is likely a result of select misclassification in the ALR (i.e., if the original treated site is recorded instead of coding the treatment as a breast boost). Overall, results of this study are consistent with previous research with other administrative databases in the US, which indicated the agreement for the receipt of radiotherapy as substantial (kappa = 0.70 to 0.79) [3, 6]. To our knowledge, this was the first paper to validate the anatomic location that received radiotherapy.

Results for chemotherapy agreement are also consistent with previous work from the US, which indicated that the agreement for the receipt of chemotherapy was substantial (kappa = 0.62 to 0.79) [3–5, 8] or almost perfect (kappa = 0.82 to 0.89) [6, 10]. Agreement was slightly lower for specific chemotherapy protocols, consistent with other research [6, 10]. Disagreement in chemotherapy protocols in our study was often a result of the ALR missing one drug from a multidrug protocol or listing no treatment information for a woman who had corresponding records in the medical charts. The latter may be explained by the limitation that some smaller hospitals in Ontario did not report to the ALR during the time period of our study [13].

Sensitivity, specificity, and PPV were high for receipt of treatment surgery, which aligns with previous research on 1991 data from the same CIHI database [11]. Agreement was lowest for axillary node dissection and sentinel lymph node biopsy, which was expected because reporting of these procedures in CIHI databases was only optional until 2015 if it occurred during the same episode as the primary lumpectomy or mastectomy. Previous research in Manitoba comparing hospital records with medical claims and the Manitoba Cancer Registry also found lower agreement for axillary node dissection when compared to other surgery types [12].

Results from this study indicated that agreement for receipt of chemotherapy is not impacted by age. This result is consistent with some literature from the US [7], but not other literature which showed a decrease in agreement with increases in age [4]. Our study also showed that chemotherapy and radiotherapy agreement increased with more recent diagnosis year. Although this finding was inconsistent with other literature [4], it was expected in our study based on active efforts to increase the accuracy of the ALR after its establishment in the late 1990s. Results from this study do align with previous research for stage, with more advanced stage of breast cancer resulting in higher odds of disagreement for receipt of chemotherapy [4]. This may suggest poorer agreement for palliative versus curative treatments. Conversely, we found that agreement increased with advanced stage of breast cancer for partial mastectomy, possibly indicating more substantial agreement for more complex cases requiring longer hospital stays.

Exact agreement in radiotherapy start dates was extremely high at 96.5%. There was slightly less agreement in radiotherapy end dates; however, the discrepancies were minor as agreement increased from 83% to 95.4% when considering end dates within 1 week of the medical chart end dates. Agreement in chemotherapy start and end dates was also high, with disagreements mostly due to missing ALR records at the beginning of treatment protocols. Agreement in dates for surgery was extremely high. Mismatched dates mostly occurred in women whose first treatment surgery was also diagnostic, as these procedures were not coded in CIHI as breast cancer-related and were therefore excluded during data cleaning. To our knowledge, only one previous study has validated dates for breast cancer treatment, finding only 86% agreement ±30 days [8]. Overall, treatment dates in the ALR and CIHI databases were highly accurate, which means that this data is being used reliably for monitoring and evaluation of Ontario treatment wait times.

Strengths of this study include a large sample size and use of data from a population-based cohort of screened women, with access to the medical charts of more than 95% of the eligible women. Also, this was the first study to our knowledge to validate breast cancer treatment types and dates in the ALR and CIHI databases. However, there are several limitations. First, the definitions used for the type of therapy may have differed between medical charts and administrative data. In addition, some types of treatments could not be compared because they were not present in both data sources. The results may not generalize to women diagnosed outside of the OBSP, those with stage IV breast cancers, in situ breast cancers, or other cancers which might have different treatments, such as oral rather than systemic chemotherapy. Finally, while medical charts were used as the gold standard, previous research has suggested that a true gold standard may not exist [27].

## 5. Conclusions

Agreement between medical charts and administrative databases for breast cancer treatment data varied from moderate to almost perfect, depending on treatment type. In future Ontario studies, chart review may not be required for collection of breast cancer treatment data. Future research could validate more specific treatment details, such as radiotherapy dose levels, to determine if administrative databases suffice for more detailed epidemiological research.

## Figures and Tables

**Table 1:** Characteristics of eligible women diagnosed with invasive breast cancer through Ontario Breast Screening Program between January 1, 2006, and December 31, 2009, by women with complete radiotherapy, chemotherapy, and surgery data.

Characteristics	Radiotherapy *N* = 2,375 *n* (%)	Chemotherapy *N* = 2,292 *n* (%)	Surgery *N* = 2,400 *n* (%)
*Age at diagnosis (years)*			
50–59	824 (34.7)	792 (34.6)	832 (34.7)
60–69	1,551 (65.3)	1,500 (65.4)	1,568 (65.3)
*Year of diagnosis*			
2006	454 (19.1)	439 (19.2)	459 (19.1)
2007	597 (25.1)	574 (25.0)	602 (25.1)
2008	613 (25.8)	593 (25.9)	618 (25.8)
2009	711 (29.9)	686 (29.9)	721 (30.0)
*Income quintile*			
1: lowest	417 (17.6)	400 (17.5)	423 (17.7)
2	466 (19.7)	447 (19.6)	471 (19.7)
3	474 (20.1)	456 (20.0)	478 (20.0)
4	447 (18.9)	432 (18.9)	452 (18.9)
5: highest	559 (23.7)	547 (24.0)	564 (23.6)
*Missing*	*12*	*10*	*12*
*Community status*			
Urban	1,962 (82.7)	1,893 (82.6)	1,985 (82.8)
Rural	132 (5.6)	129 (5.6)	133 (5.5)
Rural remote	188 (7.9)	182 (7.9)	189 (7.9)
Rural very remote	91 (3.8)	87 (3.8)	91 (3.8)
*Missing*	*2*	*1*	*2*
*Comorbidity*			
No	1,338 (57.2)	1,290 (57.2)	1,354 (57.3)
Yes	1,001 (42.8)	967 (42.8)	1,008 (42.7)
*Missing*	*36*	*35*	*38*
*Breast cancer classification*			
Invasive	698 (30.2)	675 (30.2)	703 (30.1)
Invasive + DCIS	1,615 (69.8)	1,559 (69.8)	1,635 (69.9)
*Missing *	*62*	*58*	*62*
*TNM stage*			
I	1,544 (65.9)	1,507 (66.7)	1,555 (65.7)
II	675 (28.8)	637 (28.2)	683 (28.9)
III	123 (5.3)	117 (5.2)	128 (5.4)
*Missing*	*33*	*31*	*34*
*Histologic grade*			
1	706 (30.4)	694 (30.9)	711 (30.3)
2	1,103 (47.5)	1,060 (47.2)	1,117 (47.6)
3	514 (22.1)	491 (21.9)	520 (22.1)
*Missing*	*52*	*47*	*52*
*Triple negative*			
No	2,093 (90.6)	2,034 (91.0)	2,112 (90.6)
Yes	217 (9.4)	202 (9.0)	220 (9.4)
*Missing*	*65*	*56*	*68*
*Treatment centre region*			
South Central	1,213 (51.1)	1,155 (50.4)	1,230 (51.3)
South Eastern	411 (17.3)	403 (17.6)	412 (17.2)
South Western	463 (19.5)	453 (19.8)	468 (19.5)
Northern	288 (12.1)	281 (12.3)	290 (12.1)

DCIS, ductal carcinoma in situ.

**Table 2:** Agreement analysis by receipt of treatment and specific type of treatment, comparing information from administrative databases with medical charts among women with complete radiotherapy, chemotherapy, and surgery data.

Treatment type	Medical chart *n* (%)	Administrative database^1^ *n* (%)	Sensitivity (%)	Specificity (%)	PPV (%)	NPV (%)	Kappa
*Radiotherapy*^2^							
Yes	1,970 (82.9)	1,816 (76.5)	92.0	99.3	99.8	71.9	0.793
No	405 (17.1)	559 (23.5)					
*Breast or chest wall radiation*							
Yes	1,840 (93.4)	1,735 (88.1)	88.2	n/a	93.5	n/a	n/a
No	130 (6.6)	235 (11.9)					
*Breast boost*							
Yes	510 (25.9)	230 (11.7)	43.3	99.4	96.1	83.4	0.520
No	1,460 (74.1)	1,740 (88.3)					
*Supraclavicular/axilla radiation*							
Yes	232 (11.8)	223 (11.3)	86.6	98.7	90.1	98.2	0.868
No	1,738 (88.2)	1,747 (88.7)					

*Chemotherapy*^3^							
Yes	840 (36.6)	665 (29.0)	77.7	99.2	98.2	88.5	0.804
No	1,452 (63.4)	1,627 (71.0)					
*FEC-D *							
Yes	288 (44.2)	226 (34.7)	71.9	94.8	91.6	81.0	0.682
No	364 (55.8)	426 (65.3)					
*AC *							
Yes	109 (16.7)	67 (10.3)	60.6	99.8	98.5	92.6	0.714
No	543 (83.3)	585 (89.7)					
*ACP/ACT*							
Yes	95 (14.6)	67 (10.3)	65.3	99.1	92.5	94.4	0.733
No	557 (85.4)	585 (89.7)					
*TC*							
Yes	67 (10.3)	47 (7.2)	50.7	97.8	72.3	94.5	0.559
No	585 (89.7)	605 (92.8)					
*FEC*							
Yes	48 (7.4)	32 (4.9)	62.5	99.7	93.8	97.1	0.734
No	604 (92.6)	620 (95.1)					

*Surgery* ^4^							
Yes	2,396 (99.8)	2,296 (95.7)	95.8	100	100	n/a	n/a
No	4 (0.2)	104 (4.3)					
*Partial mastectomy*							
Yes	2,035 (84.9)	1,947 (81.3)	94.2	91.7	98.5	73.7	0.781
No	361 (15.1)	449 (18.7)					
*Modified radical mastectomy*							
Yes	277 (11.6)	284 (11.9)	89.5	98.3	87.3	98.6	0.869
No	2,119 (88.4)	2,112 (88.1)					
*Simple (total) mastectomy*							
Yes	234 (9.8)	162 (6.8)	60.7	99.1	87.7	95.9	0.693
No	2,162 (90.2)	2,234 (93.2)					
*Axillary node dissection*							
Yes	818 (34.1)	244 (10.2)	27.4	98.7	91.8	72.4	0.314
No	1,578 (65.9)	2,152 (89.8)					
*Sentinel lymph node biopsy*							
Yes	1,684 (70.3)	1,441 (60.1)	73.7	71.9	86.1	53.6	0.415
No	712 (29.7)	955 (39.9)					

*Note*. Due to small numbers, values marked “n/a” could not be reliably calculated. PPV, positive predictive value; NPV, negative predictive value; FEC-D, Fluorouracil, Epirubicin, Cyclophosphamide, Docetaxel; AC, Adriamycin, Cyclophosphamide; ACP, Adriamycin, Cyclophosphamide, Paclitaxel; ACT, Adriamycin, Cyclophosphamide, Taxol; TC, Taxotere, Cyclophosphamide or Carboplatin; FEC, Fluorouracil, Epirubicin, Cyclophosphamide. ^1^The corresponding administrative database is the Activity Level Reporting database (ALR) for radiotherapy and chemotherapy treatment data, and Canadian Institute for Health Information (CIHI) databases for surgical data. ^2^Including brachytherapy/internal radiation, clinical trials, and unknown and other types of radiotherapy. *N* = 2,375 for receipt of radiotherapy (women with complete radiotherapy data in medical charts), and *N* = 1,970 for radiotherapy types (women who received radiotherapy according to medical charts). ^3^Including clinical trials, unknown, and all chemotherapy protocols. *N* = 2,292 for receipt of chemotherapy (women with complete chemotherapy data in medical charts), and *N* = 652 for chemotherapy protocols (women who received one chemotherapy treatment according to medical charts). ^4^Including partial mastectomies, modified radical mastectomies, simple (total) mastectomies, axillary node dissections, sentinel lymph node biopsies, and other surgeries. *N* = 2,400 women for receipt of surgery (women with complete surgery data in medical charts) and *N* = 2,396 for surgery type (women who received surgery according to medical charts).

**Table 3:** Odds ratios (ORs) and 95% confidence intervals (CIs) comparing agreement versus disagreement between medical records and administrative database information in women with invasive breast cancer for each treatment and its most common type.

	Radiotherapy *N* = 2,375		Breast or chest wall radiation *N* = 1,970		Chemotherapy *N* = 2,292		FEC-D *N* = 652		Partial mastectomy^2^ *N* = 2,396	
	OR^1^ (95% CI)	*p* value	OR^1^ (95% CI)	*p* value	OR^1^ (95% CI)	*p* value	OR^1^ (95% CI)	*p* value	OR^1^ (95% CI)	*p* value
*Age at diagnosis*										
50–59	1.00 (ref)		1.00 (ref)		1.00 (ref)		1.00 (ref)		1.00 (ref)	
60–69	**1.41 (1.00–1.99)**	**0.0498**	1.05 (0.82–1.35)	0.6960	1.06 (0.77–1.47)	0.7075	0.81 (0.52–1.26)	0.3512	0.81 (0.56–1.19)	0.2823
*Year of diagnosis*										
2006	1.00 (ref)		1.00 (ref)		1.00 (ref)		1.00 (ref)		1.00 (ref)	
2007	**1.59 (1.05–2.42)**	**0.0287**	0.96 (0.69–1.34)	0.8100	0.87 (0.57–1.34)	0.5323	1.05 (0.54–2.06)	0.8859	0.93 (0.53–1.64)	0.8029
2008	**1.81 (1.16–2.83)**	**0.0086**	1.04 (0.73–1.49)	0.8168	**1.94 (1.20–3.16)**	**0.0074**	1.63 (0.80–3.32)	0.1763	0.67 (0.39–1.14)	0.1418
2009	**3.72 (2.12–6.54)** ^**3**^	**<0.0001**	0.99 (0.71–1.40)	0.9766	1.49 (0.96–2.33)	0.0765	1.37 (0.69–2.73)	0.3647	0.63 (0.37–1.07)	0.0869
*TNM stage*										
I	1.00 (ref)		1.00 (ref)		1.00 (ref)		1.00 (ref)		1.00 (ref)	
II/III	1.25 (0.87–1.80)	0.2191	0.99 (0.76–1.29)	0.9637	**0.66 (0.48–0.91)**	**0.0108**	1.43 (0.87–2.32)	0.1556	**3.36 (2.27–4.99)**	**<0.0001**

*Note*. Missing values were excluded from analyses. FEC-D, Fluorouracil, Epirubicin, Cyclophosphamide, Docetaxel. ^1^Adjusted for all other variables in the table and treatment centre region. ^2^Results could not be computed for receipt of surgery overall, as there was limited variability. ^3^Test for trend *p* < 0.0001.

**Table 4:** Percent agreement between medical charts and administrative databases for radiotherapy, chemotherapy, and surgery dates.

Date validated	Exact agreement	Agreement ± 1 day	Agreement ± 7 days
Radiotherapy (breast/chest wall radiation)			
Start date	96.5%	97.5%	98.7%
End date	83.0%	84.0%	95.4%
Chemotherapy (FEC-D)			
Start date	88.7%	93.1%	96.1%
End date	85.4%	90.8%	94.7%
Surgery (partial mastectomy)	96.3%	97.2%	98.2%

ALR, Activity Level Reporting database; CIHI, Canadian Institute for Health Information; FEC-D, Fluorouracil, Epirubicin, Cyclophosphamide, Docetaxel.
